# The Double-Edged Sword Effect of Team Job Insecurity on Team Resilience

**DOI:** 10.3390/bs15101376

**Published:** 2025-10-10

**Authors:** Jingli Xue, Chunhong Liu

**Affiliations:** Glorious Sun School of Business and Management, Donghua University, Shanghai 200051, China; 1229164@mail.dhu.edu.cn

**Keywords:** team job insecurity, team challenge appraisal, team hindrance appraisal, team resilience, task interdependence

## Abstract

While previous research has examined the role of team resources on team resilience from a resource-based perspective, the underlying mechanisms of team resilience emergence from a process perspective remain insufficiently discussed. Drawing on team stress appraisal theory, we explore the mechanism through which team job insecurity influences team resilience and the contextual effects of team task characteristics. Through a three-wave questionnaire conducted with 464 employees from 96 teams, we found that team job insecurity was positively related to team challenge appraisal, which in turn was positively related to team resilience. Meanwhile, team job insecurity was positively related to team hindrance appraisal, which in turn was negatively related to team resilience. Furthermore, ream task interdependence reinforced the positive effect of team job insecurity on team resilience via challenge appraisal, while weakening the negative effect of team job insecurity on team resilience via hindrance appraisal. Both theoretical and practical contributions were discussed.

## 1. Introduction

With the increasing dynamics of the environment characterized by volatility, uncertainty, complexity, and ambiguity (VUCA, Millar et al., 2018), resilience has become more crucial for organizations (DesJardine et al., 2019; Dimitriadis, 2021; Sajko et al., 2021; Stoverink et al., 2020; Ortiz-de-Mandojana & Bansal, 2016). For example, Huawei—a renowned Chinese company—has developed a strategic reserve system, empowering young leaders to “lead the troops in battle”, which ensures the organization remains passionate and dynamic. Amazon Web Service has implemented a DevOps culture, which enhances team collaboration and ongoing improvement of team resilience. Organizational resilience refers to the adaptive capacity of an organization to actively adjust to and manage pressure under the risky context (Raetze et al., 2022), recover from crises or challenges, and even develop and transform in terms of organizational growth (Hillmann & Guenther, 2021). However, in the context of VUCA, organizations may confront significant challenges to their resilience due to the employees’ concerns about job insecurity, which reflects individuals’ perception potential threat to the stability of their present job (Jiang & Lavaysse, 2018). Unfortunately, the mechanisms and boundary conditions of how team job insecurity affects team resilience remain under-investigated.

Specifically, first, previous research exploring antecedents has focused on organizational resilience but largely overlooked team resilience (e.g., Barasa et al., 2018). Empirical studies have examined the role of leadership, organizational culture, organizational support, and psychological capital in affecting organizational resilience. However, compared to organizations, teams have fewer resources and face more pronounced employee conflicts in the uncertainty (Hobfoll et al., 2018). Thus, conclusions drawn from research on organization resilience fail to adequately apply to explaining the formation of team resilience. Although the potential negative impact of uncertainty on team resilience has been suggested (Khuan, 2024), there is a lack of empirical evidence and in-depth theoretical discussion.

Second, researchers have predominantly focused on the resource-based perspective, leaving a limited understanding of the cognitive mechanisms through which job insecurity impacts team resilience. Based on conservation of resource theory (Hobfoll, 1989), existing research has suggested that digital technologies provide teams with resources to manage uncertain events, which enhances team resilience (Miceli et al., 2021). However, in the era of VUCA, organizations frequently adjust their structures in order to respond to market changes, continuously introduce new technologies, and face high levels of uncertainty in both internal production processes and external markets. Consequently, team members typically possess a high degree of job insecurity (Wilson et al., 2020; H. J. Wang et al., 2019). In this context, on the one hand, team members may focus more on the potential growth and benefits of the challenge (Griffin & Grote, 2020) and proactively confront environmental stress, which in turn strengthens team resilience. On the other hand, they may also pay more attention to the potential losses and harms with threats (Mitchell et al., 2019), which corresponds to hindrance appraisal, provoking negative emotion-driven responses that diminish team resilience. Despite these insights, there remains a significant gap in our understanding of the mechanism of job insecurity on team resilience from the perspective of stress appraisal.

Third, further exploration is required into the contextual factors and boundary conditions of the key antecedents that affect team resilience. Employees in teams with different task characteristics may adopt different coping strategies for job insecurity (Klug et al., 2024), leading to differing levels of team resilience. Team task interdependence refers to the extent to which employees interact with or rely on other team members in terms of information, resources, and technologies to complete the tasks (Cheng et al., 2023; Courtright et al., 2015). We propose that when the level of team task interdependent is high, team members are more likely to cooperate with others to cope with the challenges or threats, which enhances team resilience. However, research on the boundary conditions through which team job insecurity affects team resilience via stress appraisal from the perspective of team task characteristics remains to be expanded.

Taken together, drawing on team stress appraisal theory, this study examines the mechanisms and boundary conditions through which team job insecurity influences team resilience via team stress appraisal. We make three key contributions to the research on team resilience. First, focusing on the VUCA context, we investigate the role of team job insecurity in shaping team resilience, thereby enriching the antecedent research of team resilience and validating the propositions of prior qualitative research (Hartwig et al., 2020). Second, based on team stress appraisal theory, we explore the team challenge appraisal and hindrance appraisal as two key mediators, fulfilling the understanding of mechanisms through which job insecurity impacts team resilience. Third, by further introducing team task interdependence, we develop a moderated mediation model to illustrate the boundary conditions under which team job insecurity affects team resilience. The conclusions of our study offer valuable insights for team leaders to deal with job insecurity and enhance team resilience.

This article contains an introduction, hypotheses, method, results, and a discussion section. In the introduction, the necessity of studying team resilience from the perspective of job security will be elaborated upon, gaps in previous research will be identified, and the research aims and contributions of this study will be outlined. In the Hypotheses section, research hypotheses will be proposed based on stress appraisal theory and prior research. The following section will describe the sampling process, the characteristics of the samples, the specific variable features, and the analytical methods employed in this study. The objective of the present investigation is to ascertain whether each hypothesis has been supported, as outlined in the results section. The subsequent discussion will present the study’s findings, theoretical contributions, practical insights, limitations, and directions for future research.

## 2. Literature Review, Research Hypotheses, and Conceptual Model

Stress appraisal theory posits that stressors do not inherently fall into distinct “positive” or “negative” categories (Lazarus, 1999; Rinker et al., 2025). Instead, it is employees’ subjective evaluations of these stressors that serve as the critical pathway influencing their motivational states and behavioral outcomes, rather than the specific stressors themselves (Gerich, 2017). As trade frictions intensify, X virus outbreaks occur, and localized conflicts erupt, the external environment of organizations continues to deteriorate. Factors such as heightened industry competition and high uncertainty surrounding market share fluctuations have led to widespread job insecurity among employees. In accordance with stress appraisal theory, team members are required to concurrently enhance their efficiency in fulfilling work tasks and improve the quality of their work in order to adapt to environmental changes. This process involves attributing a challenge-oriented stress appraisal to job insecurity, thereby continuously improving work capabilities and strengthening member collaboration, ultimately enhancing team resilience. Meanwhile, team members also address the impact of unclear career development, which prompts them to undertake a hindrance-oriented stress appraisal. This diverts their attention from their tasks and results in augmented resource consumption, consequently diminishing team resilience.

### 2.1. Team Job Insecurity, Team Challenge Appraisal, and Hindrance Appraisal

We expect that team job insecurity is positively associated with team challenge appraisal. Challenge appraisal is predominantly defined as individuals perceiving job stressors as favorable, believing they can overcome them, and that they will benefit their career progression (Webster et al., 2011). Drawing on conservation of resource theory, a study involving 415 workers in the United States found that job insecurity, insofar as it is perceived as a threat, can motivate employees to make efforts to improve themselves in order to protect themselves (Shoss et al., 2023). Thus, these individuals devote more effort to work and learn new skills to enhance their competitiveness. Previous studies have shown that complex and dynamic tasks create learning and growing opportunities for employees and invoke their curiosity, excited emotions, and job engagement (Griffin & Grote, 2020). A study of 257 employees drawn from China’s financial, sales, construction, commercial, real estate sectors, and administrative agencies was conducted. The study found that perceived job insecurity has a reinforcing effect on employees’ recognition of the need to invest greater effort in enhancing their capabilities (Yao et al., 2021). This, in turn, leads them to evaluate job insecurity as a challenging form of pressure. Therefore, we hypothesize:

**H1a.**  *Team job insecurity is positively associated with team challenge appraisal.*

We speculate that team job insecurity is also positively associated with team hindrance appraisal. A study of 419 Chinese workers in mainland China was conducted using two questionnaire surveys. The study found that hindrance appraisal is characterized by the perception of job stressors as unfavorable and the belief that they are difficult to overcome, thereby impeding career progression (Ma et al., 2021). Specifically, such individuals may be concerned about failing to meet the teams’ requirements and expectations of their teams, which in turn increases their perception of heightened hindrance-related stress from work processes, task conflict, and organizational change (Sverke et al., 2002). Furthermore, using the same methodology applied to the experiences of 108 employees, research has found that job insecurity leads individuals to pay more attention to difficulties, potential losses, and failures when they feel they have no control over their environment (Mitchell et al., 2019). A study involving 16,574 employees from 1149 companies in the UK found that when resources are insufficient to cope with job insecurity, they may experience negative emotions (W. Wang et al., 2018), thereby leading to team hindrance appraisal. A study of 459 Chinese employees found that when individuals perceive a loss of control at work, they may experience anxiety and find it difficult to accurately assess the consumption of work resources and the corresponding returns (Liu et al., 2022). Thus, we propose:

**H1b.**  *Team job insecurity is positively associated with team hindrance appraisal.*

### 2.2. Mediating Role of Team Challenge Appraisal and Hindrance Appraisal

We expect that team job insecurity will positively impact team challenge appraisal, which in turn enhances team resilience. Analyzing two-wave data from 275 employees and 58 supervisors, a study indicated that when team members perceive a high level of job insecurity, proactive individuals may realize that both opportunities and challenges are present. This mindset will motivate them to actively embrace complex tasks (Tu et al., 2024). Moreover, a study was conducted on a sample of 344 undergraduate students, which found that team members may harness energy and passion despite limited resources and time (Kavussanu et al., 2014) to deal with challenges, enhance the team’s ability to handle diverse complex situations, and achieve team goals (Kaplan et al., 2013). These efforts collectively contribute to team resilience. By using data gathered from 72 work teams and 354 individual members from 11 information and technology firms in China, research has indicated that insecurity within the team work environment could cultivate empathy and a collaborative spirit, strengthening team cohesion (Hu et al., 2018). Additionally, a two-wave study involving 91 teams (comprising 1291 individual responses) indicated that the positive emotionality within the team boosts team resilience through team reciprocity and self-reflection (Hartmann et al., 2021). Thus, we further propose:

**H2a.**  *Team challenge appraisal mediates the relationship between team job insecurity and team resilience.*

Team job insecurity may also positively influence team hindrance appraisal, which in turn decreases team resilience. When confronted with job insecurity, competition for scarce resources within the team will intensify, and data from 399 Chinese public sector employees supported the hypothesis that team members with job insecurity feel under pressure, a loss of control, and anxiety (Bao & Zhong, 2021). In contrast, team members will focus on their individual survival and performance rather than team goals, decreasing team collaborative desire and team cohesion. A two-stage questionnaire survey of 286 employees at an IT company in India was conducted, and the results of the study indicated that team job insecurity can lead employees to adopt defensive and avoidance behaviors, such as limiting information sharing and cooperation (Nifadkar & Bauer, 2016). A questionnaire survey of 306 hospital staff at a large public hospital located in Iran revealed that hindrance stressors can significantly influence negative emotions, such as anxiety and worry, as well as emotional exhaustion (Charkhabi, 2019), and a study of 192 American managers and 271 Italian blue-collar workers found that anxiety can diminish team members’ organizational identification, and promote counterproductive behaviors (Piccoli et al., 2021). Therefore, we hypothesize:

**H2a.**  *Team hindrance appraisal mediates the relationship between team job insecurity and team resilience.*

### 2.3. Moderating and Moderated Mediating Effects of Team Task Interdependence

We further expect that team task interdependence is an important contextual variable in the indirect relationship between team job insecurity and team resilience. When task interdependence is high, team members realize that they cannot complete the task solely by relying on the information and resources at their disposal. The results of a meta-analysis study indicate that team members in groups with a high level of task interdependence must rely on each other’s expertise, information, and resources to collaborate in addressing challenging tasks and sharing team results (Courtright et al., 2015). Therefore, they will proactively engage in interactions and contacts among team members (e.g., Courtright et al., 2015), promoting team performance (Widianto et al., 2024).

A study of 386 athletes at a Canadian university indicated that, in the face of job insecurity, teams perceive their environment as offering opportunities to build competitive advantage, such as developing new core competencies and enhancing their capabilities, and thus have a high level of challenge pressure and tend to invest more effort in improving the team’s competitiveness (e.g., Wolf et al., 2015). A study of 133 teams from three Spanish savings banks indicated that when the level of team task interdependence is high, team members perceive the improvement of the team’s overall competence as the basis for the realization of their self-interests, and thus tend to take the initiative to work for the improvement of the team (e.g., Le Blanc et al., 2021), in this case, organizations are more likely to gather resources and synchronize actions to confront stressors and emergencies, demonstrating a high level of resilience (Kahn et al., 2018), leading to the strengthening of team resilience. In support of our proposal, a meta-analysis based on 415 effect sizes from 69 studies has indicated that task interdependence increases the positive attitude towards team members (Ryu et al., 2022). Thus, we propose:

**H3a.**  *Team task interdependence moderates the relationship between team challenge appraisal and team resilience. Specifically, when the level of team task interdependence is high, this relationship will be stronger.*

**H3b.**  *Team task interdependence moderates the indirect effect of team job insecurity on team resilience via team challenge appraisal. Specifically, when the level of team task interdependence is high, this indirect effect will be stronger.*

In the face of job insecurity, team members recognize the scarcity of resources and fear that they will not be able to achieve the team’s goals, resulting in a loss of interest among members, which generates negative emotions and thus higher ratings of obstructive stress. At this point, when team task interdependence is high, team members recognize that strengthening team communication and improving team performance are key ways to avoid negative outcomes, team members will alter their negative and avoidant attitudes, engage in collaboration with each other, and effectively reduce the burden of individual work pressure and the need to deal with difficult work issues (LePine et al., 2005). These actions further boost the team’s ability to handle difficulties and its overall resilience (Barton & Kahn, 2019; Varajão et al., 2021). Existing research has suggested that resilient team members are better able to handle stress (Varajão et al., 2021) and promote team performance (Van der Beek & Schraagen, 2015) when work problems are highly complex and likely to cause conflict (Varajão et al., 2021). Thus, we hypothesize:

**H4a.**  *Team task interdependence moderates the relationship between team hindrance appraisal and team resilience. Specifically, when the level of team task interdependence is high, this relationship will be weaker.*

**H4b.**  *Team task interdependence moderates the indirect effect of team job insecurity on team resilience via team hindrance appraisal. Specifically, when the level of team task interdependence is high, this indirect effect will be weaker.*

**Figure 1:**
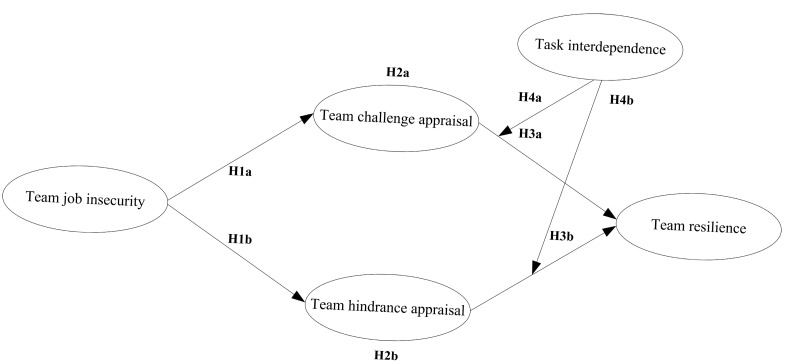
Hypothesized model.

The full model is as follows (See Figure 1).

## 3. Method

The present study focuses on the mechanism by which job insecurity influences team resilience. In order to enhance external validity, a questionnaire-based research method was adopted in order to quantitatively validate how team job insecurity affects team resilience through two stress appraisals, as well as the impact of task interdependence.

### 3.1. Samples and Procedures

Five hundred and thirty-two employees of the first-line 114 production team of a manufacturing group company in a province on the east coast of China participated in this study. The city’s manufacturing industry, which relies predominantly on foreign trade, has experienced significant challenges due to the deterioration of the trade situation. This has led to a notable decline in sales volume, causing widespread feelings of job insecurity among the production team working at the front line. Consequently, the sample of the study demonstrated a high degree of alignment with the research question. The data collection process of the present study can be divided into the following steps: firstly, the random selection of the study sample. We first obtained the support of the company to get the list of employees from the Human Resources Department and randomly selected the sample from them. Secondly, the purpose of the study was made clear. The subjects were informed that the content of the questionnaire pertained to the survey team’s resilience, that its completion is intended to contribute to the development of a scientific research paper, and that the results of the questionnaire will be utilized exclusively for academic research. It was emphasized that the questionnaire is designed to provide an overall analysis and will not disclose any personal information or be associated with individual salaries or performance evaluations. The ultimate sample size is contingent upon the employees’ consent to participate in the study. Thirdly, a randomized questionnaire was conducted. In order to address any issues that might arise during the questionnaire process, two supervisors from human resources and two members of the research team collaborated on the entire questionnaire distribution and collection process.

In order to effectively avoid common method bias (Podsakoff et al., 2003), this study conducted a three-wave time-lagged data collection. Specifically, at the first time point, employees were asked to report their job insecurity, as well as their demographic variables. Two weeks later, at the second time point, employees were asked to report their challenging and hindrance appraisal, and task interdependence. Finally, two weeks later, at the third time point, employees were asked to report perceived team resilience. The present study was a team-level study and, therefore, the individual-level measures of perceived job insecurity, challenging and hindrance appraisal, perceived task interdependence, and perceived team resilience were ultimately aggregated.

During the first round of data collection, we distributed a total of 532 questionnaires to 114 teams, and 511 questionnaires were returned (106 teams, response rate 96.05%). In the second round of data collection, we distributed 511 questionnaires (106 teams), and 486 questionnaires were returned (103 teams, response rate 95.10%). In the third round of data collection, we distributed 486 questionnaires (103 teams), and 464 questionnaires were returned (96 teams, response rate 95.47%). Due to the effective controlling in the questionnaire process, the questionnaire recovery rate was relatively high, and the final valid questionnaires were 464 (96 teams) after matching. In the sample of this study, team sizes ranged from 3 to 6 members, with an average of 4.83 members per team. The male employees were 199 (42.9%); the average age of the sample was 35.11 years (*SD* = 9.61). There were 330 employees with high school education and above (71.10%); the average tenure of the sample was 9.12 years (*SD* = 7.53).

### 3.2. Measures

In this study, all measures were administered in Chinese (Table 1), following the translation/back-translation process to maintain their validity (Brislin, 1980). To avoid common method bias, the measurement of all variable items was conducted utilizing a 5-point Likert scale rating method (1 = *strongly disagree*, 5 = *strongly agree*), with the exception of job insecurity and task interdependence items, which were evaluated through a 7-point Likert scale (1 = *strongly disagree,* 7 = *strongly agree*).

***Job Insecurity.*** We used De Witte’s four-item Job Insecurity Scale (De Witte, 2000; Vander Elst et al., 2014) to measure employees’ job insecurity (α = 0.75). Sample question items such as “I feel insecure about the future of my job” and aggregation statistics supported aggregating to the team level: ICC1 = 0.15, ICC2 = 0.46; the mean of rwg (j) = 0.75 (James et al., 1984).

***Challenge and hindrance appraisals.*** We used items from Rodell and Judge’s scale (Rodell & Judge, 2009) to measure employees’ challenge and hindrance appraisal (α = 0.74). Three of the items were used to measure challenging appraisal, with sample items such as “My job has required me to use a number of complex or high-level skills”. The aggregation statistics supported aggregating to the team level: ICC1 = 0.16, ICC2 = 0.47; the mean of rwg (j) = 0.86. The other three items were used to measure hindrance appraisal (α = 0.82), with sample items such as “I have not fully understood what is expected of me”, and the results of aggregation statistics supported aggregating to the team level: ICC1 = 0.34, ICC2 = 0.72; the mean of rwg (j) = 0.82.

***Team resilience.*** We used three items developed by Kantur and Say (2015) to measure team resilience (α = 0.83). Sample question items such as “we develop alternatives in order to benefit from negative circumstances”, and the aggregation statistics supported aggregating to the team level: ICC1 = 0.32, ICC2 = 0.70; the mean of rwg (j) = 0.86.

***Task interdependence.*** We used a five-item scale developed by Van der Vegt and Janssen (2003) to measure task interdependence (α = 0.79). Sample items such as “My colleagues need information and advice from me to perform their jobs well, and the aggregation statistics supported aggregating to the team level: ICC1 = 0.48, ICC2 = 0.82; the mean of rwg (j) = 0.80.

## 4. Results

### 4.1. Data Analysis Strategy

Due to the team-level nature of this study, we firstly aggregated the individual-level variable measures into the team level. Secondly, by using Mplus7.0 (Muthén & Muthén, 2017), we tested the indirect effect of team job insecurity on team resilience via team challenge appraisal and team hindrance appraisal, respectively. Third, we conducted a Monte Carlo sampling method to estimate the confidence intervals for these indirect effects (Preacher et al., 2010). Finally, we tested the moderating effects of task interdependence, plotted the simple slope of this moderation effect, and tested the moderated mediation effect of task interdependence, and conducted a Monte Carlo sampling method to estimate the confidence intervals for these moderated mediation effects.

We conducted a conformative factor analysis by using Mplus 7.0 (Muthén & Muthén, 2017). The results of the five-factor model fit well (*χ*^2^ = 266.96, *df* = 125, *p* < 0.00, *CFI* = 0.95, *TLI* = 0.93, *RMSEA* = 0.05, *SRMR* = 0.04), and better than alternative model (four-factor model, collapsing challenge appraisal and hindrance appraisal, *χ*^2^ = 755.63, *df* = 129, *p* < 0.00, *CFI* = 0.76, *TLI* = 0.72, *RMSEA* = 0.10, *SRMR* = 0.09; three-factor model, collapsing team challenge appraisal, team hindrance appraisal, and team resilience, *χ*^2^ = 1035.22, *df* = 132, *p* < 0.00, *CFI* = 0.66, *TLI* = 0.60, *RMSEA* = 0.12, *SRMR* = 0.11, which provided evidence that the variables in this research had good discriminant validity. The means, variances, and correlations of the variables are displayed in Table 2. The findings indicated a positive correlation between team job insecurity and challenging appraisal (*r* = 0.30, *p* < 0.01) and team hindrance appraisal (*r* = 0.27, *p* < 0.01). Further, team challenge appraisal was positive with team resilience (*r* = 0.43, *p* < 0.01), and team hindrance appraisal was negative association with team resilience (*r* = −0.38, *p* < 0.01), which basically supports the hypotheses in this study.

### 4.2. Mediation Effects Test

Hypotheses 1a and 2a suggest that team job insecurity is positively associated with team challenge appraisal, and team challenge appraisal mediates the indirect effect of team job insecurity on team resilience. The results^1^ showed that team job insecurity was positively related to team challenge appraisal (*β* = 0.22, *p* < 0.01, see Table 3), and that the indirect effect of team job insecurity on team resilience via team challenge appraisal was significant (0.13, 95% CI (0.028, 0.227)). By using of Monte Carlo simulation method (Preacher et al., 2010), the results showed that the 95% confidence interval (20,000 repetitions) for this indirect effect did not contain zero (CI (0.037, 0.237)), thus, Hypothesis 1a and Hypothesis 2a were supported.

Based on the same test procedure, the results indicated that team job insecurity was positive related to team hindrance appraisal (*β* = 0.31, *p* < 0.05, see Table 2), and that the indirect effect of team job insecurity on team resilience through team hindrance appraisal was significant (−0.12, 95% CI (−0.220, −0.021)); therefore, Hypothesis 1b and Hypothesis 2b were supported.

### 4.3. Moderation Effect and Moderated Mediation Effect Test

**Figure 2:**
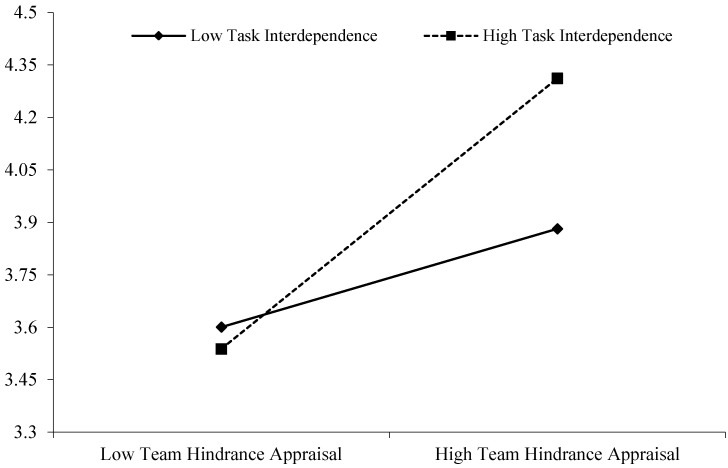
Interactive effects of the team challenge appraisal and task interdependence on team resilience.

Hypothesis 3a argues that task interdependence moderates the relationship between team insecurity and team challenge appraisal, and moderates the relationship between team insecurity and team hindrance appraisal. The results showed that the effect of team job insecurity on team challenge appraisal was positively significant (0.80, *SE* = 0.17, 95% CI (0.477, 1.124), see Table 3, Figure 2) for teams with high level of task interdependence (+1 *SD*), however, this effect was non-significant (0.29, *SE* = 0.18, 95% CI (−0.062, 0.644)) for team with low level of task interdependence (−1 *SD*), and there was a significant difference in the relationship between team job insecurity on team challenge appraisal when task interdependence were high versus low (0.51, *SE* = 0.23, 95% CI (0.052, 0.968)). The Monte Carlo simulations showed that the confidence interval for this indirect effect does not contain zero (20,000 repetitions, 95% CI (−0.233, −0.031)), thus, Hypothesis 3a was supported.

**Figure 3:**
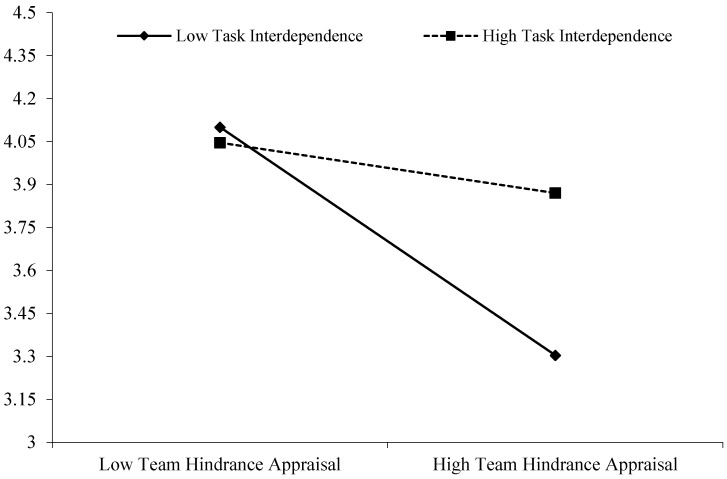
Interactive effects of the team hindrance appraisal and task interdependence on team resilience.

The results indicated that the effect of team job insecurity on team hindrance appraisal was positively significant (−0.54, *SE* = 0.10, 95% CI (−0.728, −0.349), see Table 4, Figure 3) for teams with low level of task interdependence (−1 *SD*), however, this effect was non-significant (−0.20, *SE* = 0.11, 95% CI (−0.423, 0.015)) for team with high level of task interdependence (+1 *SD*), and there was a significant difference in the effect of team job insecurity on team hindrance appraisal when task interdependence were high versus low (0.34, *SE* = 0.14, 95% CI (0.062, 0.609)), therefore, Hypothesis 4a was supported.

Hypotheses 3b and 4b propose that task interdependence enhances the indirect effect of team insecurity on team resilience through team challenge appraisal, and enhances the indirect effect of team insecurity on team resilience via team hindrance appraisal. The results indicated that the indirect effect of team insecurity on team resilience via team challenge appraisal was non-significant (*γ* = 0.11, *SE* = 0.06, 95% CI (−0.012, 0.234)). Specifically, this indirect effect was significant for teams with a high level of task interdependence (+1 *SD*, *γ* = 0.18, *SE* = 0.07, 95% CI (0.042, 0.307), see Table 3), however, this indirect effect was non-significant for teams with low level of task interdependence (−1 *SD*, *γ* = 0.06, *SE* = 0.04, 95% CI (−0.024, 0.151)). However, the Monte Carlo simulations showed that the confidence interval for this moderated effect does not contain zero (20,000 repetitions, 95% CI (0.005, 0.193), supporting Hypothesis 3b.

Meanwhile, the indirect effect of team insecurity on team resilience via team hindrance appraisal was non-significant (*γ* = 0.11, *SE* = 0.06, 95% CI (−0.009, 0.220), see Table 3), for teams with a low level of task interdependence (−1 *SD*, *γ* = −0.17, *SE* = 0.07, 95% CI (−0.304, −0.034), see Table 4), however, this indirect effect was non-significant for teams with high level of task interdependence (+1 *SD*, *γ* = −0.06, *SE* = 0.04, 95% CI (−0.147, 0.019)). However, the Monte Carlo simulations showed that the confidence interval for this moderated effect does not contain zero (20,000 repetitions, 95% CI (0.008, 0.182)), supporting Hypothesis 4b.

## 5. Discussion

Derived from a questionnaire survey conducted on 96 teams and 464 employees, we conclude that team job insecurity can increase team challenge appraisal, which in turn enhances team resilience. Meanwhile, team job insecurity can disrupt team resilience through team hindrance appraisal. Furthermore, team task interdependence strengthens the positive relationship between challenge appraisal and team resilience, while mitigating the negative correlation between hindrance appraisal and team resilience. These findings have several important theoretical contributions and practical implications.

### 5.1. Theoretical Contributions and Practical Implications

First, in the context of the VUCA era, this study examines the impact of team job insecurity on team resilience, thereby advancing research on the antecedents of team resilience. Previous studies have explored the antecedents of organizational resilience, such as team resources (Hartwig et al., 2020) and team structure (Chapman et al., 2020). However, unlike organizations, teams have fewer resources and focus more on interpersonal interactions. Therefore, the factors that contribute to team resilience may differ significantly from those that contribute to organizational resilience; there has been limited focus on the antecedents of team resilience (Barasa et al., 2018). Especially, as global competition intensifies, market demands shift rapidly, and unexpected crises frequently impact organizations, team members are generally experiencing a high level of job insecurity. In the context of the VUCA era, this study focuses on the above issues and examines the relationship between team job insecurity and team resilience. Therefore, compared to previous studies, this research expands upon the existing body of work on the antecedents of team resilience, offering a fresh perspective on the subject.

Second, focusing on team processes, we reveal the mechanism through which team job insecurity influences team resilience. From a resource perspective, certain studies have investigated the mechanisms through which organizations may enhance their resilience. Such mechanisms include, but are not limited to, digitalization, which has been demonstrated to improve the efficiency of resource orchestration within an organization (Xie et al., 2023), and social networks, which have been shown to augment an organization’s resource supply, thereby enhancing its resilience (Barasa et al., 2018). In teams, interpersonal interaction emerges as a critical factor influencing team performance (Lehmann-Willenbrock, 2025). However, research on its role in generating resilience remains limited. We introduce team stress appraisal theory to examine the diverse effects of team job insecurity on team resilience, which deepens our understanding of the mechanisms through which team job insecurity affects team resilience. Specifically, our findings show that team job insecurity enhances team resilience via team challenge appraisal, while decreasing team resilience via team hindrance appraisal. This conclusion expands upon previous research based on motivation logic, with scholars suggesting that team job insecurity reduces work engagement (Lee et al., 2024), thereby diminishing team resilience. Therefore, our study contributes to exploring the “black box” of the process through which job insecurity affects team resilience.

Third, by showing the moderating effect of team task interdependence, we broaden the understanding of the boundary conditions that influence the effect of team stress appraisal on team resilience. Research focusing on leadership characteristics has shown that leaders with a performance goal orientation reduce the positive effect of team voice on team resilience (Fisher et al., 2023). Additionally, studies examining team climate characteristics suggest that a diverse leadership environment can amplify the influence of inclusive leadership on team resilience (Hundschell et al., 2025). For teams, however, team performance is the result of team members collaborating with each other around work processes (Van der Voet & Steijn, 2021). The interdependence of tasks may have a substantial impact on team members’ interpretations of job insecurity, which in turn may influence the relationship between job insecurity and team resilience (Feng et al., 2023). Therefore, we focus on team task characteristics, with findings showing that team task interdependence enhances the positive relationship between team job insecurity and team resilience via team challenge appraisal, while decreasing the negative relationship between team job insecurity and team resilience via team hindrance appraisal. Therefore, our study expands the explanatory power of team resilience and advances our knowledge of the contextual mechanisms through which team job insecurity affects team resilience.

### 5.2. Practical Implications

The current research also provides important practical implications. First, our study finds that team challenge appraisal exerts a positive effect on team resilience, while team hindrance appraisal negatively impacts team resilience. Therefore, team managers can foster challenging stress by increasing employees’ job responsibilities through work design. These practices can heighten team members’ challenge appraisal while diminishing hindrance appraisal, which in turn enhances team resilience. For example, the team can provide training to help employees cope with stress (Gamaiunova et al., 2024). The team can also listen to employees’ problems at work and help them with their work (Lu et al., 2023). This will help employees feel able to complete their work tasks and cope with challenges stress at work. It will also help them deal with work stress better. At the same time, the teams can open a work stress consulting service for team members to reduce their hindrance stress appraisal (Kundi et al., 2022). The teams can do a good job of work planning for team members to improve their time use efficiency, which in turn reduces their evaluation of hindrance stress appraisal (Ali & Khan, 2025), and thus improves the team’s work resilience.

Second, in the context of the VUCA era, managers should pay more attention to team members’ job insecurity, ensuring employees realize that the influence of job insecurity can be shaped by individual cognition. Managers can maximize the positive effect of team job insecurity by promoting team members’ efficacy and perception of support, such as involving them in decision-making processes, offering timely feedback on their work, and providing resources that continuously improve their willingness and ability to collaborate. For example, make employees see job insecurity as a result of economic insecurity and tell them that the way to reduce job insecurity is to improve themselves. This will make them want to take the initiative to learn and enrich their own knowledge, such as knowledge of the use of digital tools. It will also help them to improve their abilities (Meneghel et al., 2016), such as searching, analyzing, identifying opportunities, and coping with adversity. This, in turn, strengthens the resilience of the team.

Third, managers can enhance the positive impact of team job insecurity on team resilience by redesigning jobs to promote team task interdependence. For example, managers can encourage team collaboration by strengthening the connections between team tasks and setting team-based performance indicators. For example, the organization can make the work publicity stronger (Chen et al., 2021). This will help employees to understand that the interests of team members are the same. They will realize that they need to work together to deal with problems, such as sharing information and helping each other to solve work difficulties. The organization also set up team-based performance appraisal measures (Keegan & Meijerink, 2023). This included indicators such as the team’s completion rate of team tasks and the atmosphere of teamwork. At the same time, some activities that are done as a team can be included in daily sports activities (Wen & Wu, 2025). This helps to make sure that everyone has the same idea of what their job is and makes the team members rely on each other more.

### 5.3. Limitations and Future Directions

Although our study contributes to the initial exploration of the antecedents, mechanisms, and boundary conditions of team resilience, several limitations to the present study should be noted. First, there may exist alternative mechanisms that explain the effect of team job insecurity on team resilience, such as resource conservation theory. Team job insecurity may deplete substantial cognitive resources from team members in their daily tasks. According to the conservation of resources theory (Halbesleben et al., 2014), to preserve essential resources for dealing with unexpected events, employees may engage in knowledge-hiding behaviors, which lower team innovation and, consequently, negatively impact team resilience. Therefore, future research could explore the double-edged sword effect of job insecurity on team resilience based on the conservation of resource theory.

Second, this study identifies the moderating role of team task interdependence from the perspective of task characteristics, while other boundary conditions remain to be explored. For example, leadership traits may be another important perspective. Transformational leadership has a role-model effect, and when employees observe their leaders engaging in job crafting, they are likely to imitate this behavior (Hetland et al., 2018). Employees’ cognitive job crafting can change their cognition of their work stress, thereby influencing the effect of job insecurity on diverse appraisal processes. Consequently, future research could explore the contextual impact of transformational leadership on teamwork insecurity on team resilience, as well as the impact of employee perceptions on the aforementioned relationships.

## 6. Conclusions

Using a three-wave survey design, the current research demonstrates that team challenge appraisal mediates the positive effect of team job insecurity on team resilience, while team hindrance appraisal mediates the negative effect of team job insecurity on team resilience. Team task interdependence plays a positive role throughout the entire mechanism. Specifically, team task interdependence strengthens the positive indirect effect of team job insecurity on team resilience via team challenge appraisal, while weakening the negative indirect effect of team job insecurity on team resilience via team hindrance appraisal. These findings highlight the significance of managing team job insecurity in the VUCA environment and offer valuable managerial insights for fostering team resilience. It is recommended that future research focus on the VUCA context and investigate the antecedents and mechanisms of team resilience within specific organizational settings, including those in safety-critical industries. Alternatively, the investigation may concentrate on management practices to explore the contextual factors influencing the relationship between team members’ job insecurity and team resilience.

## Figures and Tables

**Table 1:** Survey items.

Variables	Items	Factor Loading	Source
**Job Insecurity** **(α = 0.75)**	1. Chances are, I will soon lose my job	0.93	De Witte (2000) and Vander Elst et al. (2014)
	2. I am sure I can keep my job	0.95	
	3. I feel insecure about the future of my job	1.00	
	4. I think I might lose my job in the near future	0.72	
**Team Challenge Appraisal** **(α = 0.74)**	1. My job has required me to work very hard.	0.88	Rodell and Judge (2009)
	2. I’ve felt the weight of the amount of responsibility I have at work.	1.00	
	3. My job has required me to use a number of complex or high-level skills.	0.79	
**Team Hindrance Appraisal** **(α = 0.82)**	1. I have had to go through a lot of red tape to get my job done.	0.86	
	2. I have had many hassles to go through to get projects/assignments done.	0.93	
	3. I have not fully understood what is expected of me.	1.00	
**Team Resilience** **(α = 0.83)**	1. We develop alternatives in order to benefit from negative circumstances.	1.00	Kantur and Say (2015)
	2. We are successful in generating diverse solutions to negative circumstances.	0.92	
	3. Our team is a place where all the employees are engaged to do what is required of them.	0.91	
**Task Interdependence** **(α = 0.79)**	1. I need information and advice from my colleagues to perform my job well	0.87	Van der Vegt and Janssen (2003)
	2. I have a one-person job; it is not necessary for me to coordinate or cooperate with others	0.56	
	3. I need to collaborate with my colleagues to perform my job well	0.84	
	4. My colleagues need information and advice from me to perform their jobs well	1.00	
	5. I regularly have to communicate with colleagues about work-related issues	0.92	

**Table 2:** Means, standard deviations, and correlations.

Variables	*M*	*SD*	1	2	3	4	5
1. Team job insecurity	4.50	0.66	*(0.75)*				
2. Team challenge appraisal	3.66	0.48	0.30 **	*(0.74)*			
3. Team hindrance appraisal	3.07	0.77	0.27 **	−0.05	*(0.82)*		
4. Team resilience	3.84	0.66	0.12	0.43 **	−0.38 **	*(0.83)*	
5. Task interdependence	4.27	0.93	−0.11	0.03	0.04	0.17	*(0.79)*

*Note: N* = 464, *n* = 96. Cronbach’s alphas are in the parentheses on the diagonal. ** *p* < 0.01, two–tailed.

**Table 3:** Indirect effects analysis.

Variables	Challenge Appraisal	Hindrance Appraisal	Team Resilience	
Team job insecurity	0.22 ** (0.07)	0.31 ** (0.12)	−0.01 (0.10)	0.24 * (0.10)
Challenge appraisal			0.58 ** (0.13)	
Hindrance appraisal				−0.38 ** (0.08)
Indirect effect		Bootstrap, 95% CI	Monte Carlo, 95% CI, 20,000 repetitions	
Team job insecurity → Challenge appraisal → Team resilience		0.13 * (0.028, 0.227)	(0.037, 0.237)	
Team job insecurity → Hindrance appraisal → Team resilience		−0.12 * (−0.220, −0.021)	(−0.233, −0.031)	

*Note: N* = 464, *n* = 96; * *p* < 0.05. ** *p* < 0.01; CI = confidence interval.

**Table 4:** Moderation effects and moderated mediation effects analysis.

Dependent Variable	Moderator Task Interdependence	Effect 1 (*P_M1X_*)	Effect 2 (*P_YM1_*)	Effect (*P_M1X_* × *P_YM1_*)	95% CI of Indirect Effect, Bootstrap	95% CI of Indirect Effect, 20,000 Repetitions, Monte Carlo
Team resilience	Low (−1 *SD*)	0.22 ** (0.07)	0.29 (0.06)	0.06 (0.04)	(−0.024, 0.151)	(0.008, 0.178)
	High (+1 *SD*)		0.80 ** (0.17)	0.18 * (0.07)	(0.042, 0.307)	(0.051, 0.298)
	Diff		0.51 * (0.23)	0.11 (0.06)	(−0.012, 0.234)	(0.005, 0.193)
Dependent variable	Moderator Task interdependence	Effect 1 (*P_M2X_*)	Effect 2 (*P_YM2_*)	Effect (*P_M2X_* × *P_YM2_*)	95% CI of indirect effect, bootstrap	95% CI of indirect effect, 20,000 repetitions, Monte Carlo
Team resilience	Low (−1 *SD*)	0.31 ** (0.12)	0.54 ** (0.10)	−0.17 * (0.07)	(−0.304, −0.034)	(−0.299, −0.040)
	High (+1 *SD*)		−0.20 (0.11)	−0.06 (0.04)	(−0.147, 0.019)	(−0.172, −0.010)
	Diff		0.34 * (0.14)	0.11 * (0.06)	(−0.009, 0.220)	(0.008, 0.182)

*Note: N* = 464, *n* = 96; * *p* < 0.05. ** *p* < 0.01; *P_M1X_* refers to the effect of team challenge appraisal on team job insecurity, *P_YM1_* refers to the effect of team resilience on team challenge appraisal; *P_M1X_* × *P_YM1_* refers to the indirect effect of team job insecurity on team resilience via team challenge appraisal. *P_M2X_* refers to the effect of team hindrance appraisal on team job insecurity, *P_YM2_* refers to the effect of team resilience on team hindrance appraisal; *P_M2X_* × *P_YM2_* refers to the indirect effect of team job insecurity on team resilience via team hindrance appraisal. Diff refers to the indirect effect difference between a high level and a low level of task interdependence. CI = confidence interval.

## Data Availability

The data that support the findings of this study are available from the corresponding author upon reasonable request.

## Note

1 This study tried to control for team size as a variable and found that team size did not have a big effect on the results. For simplicity’s sake, we will no longer be reporting the influence of team size.
